# Uncommon diagnostic aspects of adolescent endometriosis: case series with narrative review of the literature

**DOI:** 10.3389/frph.2025.1700299

**Published:** 2026-01-12

**Authors:** Robert Peterek, Karolina Kowalczyk, Aleksandra Leziak, Rafał Stojko, Agnieszka Drosdzol-Cop

**Affiliations:** 1 Department of Gynecological Endocrinology, Faculty of Medical Sciences in Katowice, Medical University of Silesia, Katowice, Poland; 2 Department of Gynecology, Obstetrics and Gynecological Oncology, Faculty of Health Science in Katowice, Medical University of Silesia, Katowice, Poland

**Keywords:** endometriosis, adolescents, laparoscopy, atypical presentation, bowel lesions, hydrosalpinx, coexisting ovarian tumor, pelvic organs shift

## Abstract

**Objective:**

Endometriosis is a gynecological disorder for which awareness and detection rates are rising globally. The purpose of this study is to highlight atypical presentations of the disease; to present four adolescent patients with laparoscopically confirmed endometriosis at our hospital in Katowice, Poland, in 2024; and to discuss these cases in the context of the relevant literature.

**Methods:**

We present the clinicopathological data of four adolescent patients with atypical presentations of endometriosis who were treated at our institution with a comprehensive report of laparoscopic examination. We performed a narrative PubMed review for the period 2017–2025 to identify and discuss atypical clinical symptoms and laparoscopic findings of adolescent endometriosis. We used the following keywords were used: endometriosis, adolescents, laparoscopy, atypical presentation, bowel lesions, hydrosalpinx, coexisting ovarian tumor, pelvic organs shift. Inclusion criteria were adolescent age of patients (age 10–19) and laparoscopic confirmation of endometriosis diagnosis. We excluded articles published in languages other than English.

**Results:**

Young patients present atypical, non-specific clinical symptoms and diverse laparoscopic appearances. Shared features included lesions on the serous membrane of the large bowel and pelvic structural distortion as a consequence of uterosacral ligaments shortening or uterine adherence to the anterior abdominal wall. Additional findings included hydrosalpinx and the coexistence of an ovarian tumor. The follow-up confirmed the therapeutic success as a consequence of compliance with the oral contraceptive regimen and the following laparoscopic approach, despite the moderate-stage endometriosis in the majority of the reported cases.

**Conclusions:**

In adolescents, the diagnosis of endometriosis remains challenging because of non-classical clinical manifestations and atypical laparoscopic findings compared with those observed in adults. The presented atypical cases underscore the importance of careful interview and consideration of rare anatomical anomalies that may co-occur with endometriosis. Increasing awareness of the disease's diverse clinical variants is important among adolescent patients, their parents, and especially pediatricians, who are often the first medical contact for these patients.

## 1 Introduction

Endometriosis is a gynecologic disease associated with the presence of endometrial glands and stroma outside the uterus (1). The pathogenesis of endometriosis is related to inflammation and estrogen-dependent mechanisms (2). Aromatase and estrogen receptors are abundantly expressed in endometriotic tissue, contributing to the increased estrogen production, which in turn stimulates tissue growth (3). Pain symptoms are related to an increase in prostaglandin production, stimulated by local estrogen production via aromatase activity, together with estrogen produced by the ovaries (2). Endometriosis is estimated to affect approximately 10%–15% of females. In adolescents, prevalence appears similar, although the issue is possibly underreported in both groups (1).

According to statistics, 25%–38.3% of teenage girls with persistent pelvic pain have endometriosis (4, 5). Moreover, endometriosis is recognized as the main cause of secondary dysmenorrhea in teenagers and young adults (2). The true prevalence of endometriosis in girls remains uncertain; however, approximately two-thirds of teens with dysmenorrhea or persistent pelvic pain unresponsive to hormonal treatment and non-steroidal anti-inflammatory drugs (NSAIDs) are diagnosed with endometriosis during diagnostic laparoscopy (6, 7). Expert-performed ultrasound examinations are less commonly used in the pediatric population compared with adults, primarily because the transvaginal ultrasound approach is infrequently applied (8, 9).

Among adolescents, clinical manifestations are atypical and may be more distinct than in adults (10, 11). Deep endometriosis (DE) has a rare presentation in adolescents (12). Symptomatology in teenagers is typically defined by chronic pelvic pain and dysmenorrhea, non-cyclic pain, dyspareunia, urinary and gastrointestinal symptoms (13), low back pain, abdominal discomfort, heavy menstrual bleeding, dizziness, headaches, fatigue, and behavioral issues (14, 15). Moreover, depression and anxiety are significantly more frequent in young women with dysmenorrhea (16). These health issues may impair daily life, making it challenging for young girls to learn and engage in physical activities (17). Positive family history, early menarche, and coexistence of Müllerian abnormalities are possible risk factors for endometriosis in adolescents (18). According to the American College of Obstetricians and Gynecologists (ACOG) (2018) and the European Society of Human Reproduction and Embryology (ESHRE) (2022) guidelines, the diagnostic process involves an interview, pain diary assessment, and gynecological examination with Tanner's sexual maturity assessment. In cases of dysmenorrhea, hormonal therapy in combination with NSAIDs is recommended. First-line hormonal treatment for endometriosis in adolescents includes combined oral contraception or progestogens—dienogest 2 mg daily for 9–12 months—or levonorgestrel-releasing intrauterine system (LNG-IUS), as both options are considered safe and effective. Additionally, to treat endometriosis-associated pain, NSAIDs and other analgesics may be prescribed. Gonadotropin-releasing hormone (GnRH) agonists are prescribed as second-line therapy due to their side effects on bone mineralization and are always combined with estrogen add-back therapy. When pain management and first- and second-line treatment fail, diagnostic laparoscopy is recommended and considered the last diagnostic step (10, 13, 19). To confirm endometriosis, endometriotic lesions in laparoscopy or positive histopathological results are needed; however, negative histopathological results do not rule out the condition (10, 13).

## 2 Case description and diagnostic assessment

All the patients who qualified for surgery did not respond to 6 months of treatment with NSAIDs and combined oral contraception. None of the patients included in the study had a family history of endometriosis, and all were previously healthy, with no comorbidities. A gynecological examination was performed on each patient to exclude an obstructive anomaly. After abdominal ultrasound and pelvic MRI yielded negative or inconclusive results, the patients were qualified for diagnostic laparoscopy to establish the diagnosis. Many patients reported persistent pain despite standard management. Some adolescent patients also experienced depressive or anxiety symptoms. Other frequently noted symptoms included painful bowel movements, nausea, and urinary issues, particularly during menstruation. Blood test results were typically within normal limits. For the assessment of endometriosis, the revised American Society for Reproductive Medicine (rASRM) (20) and #ENZIAN (21, 22) scoring systems were used. rASRM categorizes endometriosis into four stages based on the number, depth, size, and location of lesions: minimal (stage I) with 1–5 points, mild (stage II) with 6–15 points, moderate (stage III) with 16–40 points, and severe (stage IV) with over 40 points (20). The #ENZIAN classification, used to assess DE, is based on the initial letters of the names of the affected organs (P, peritoneum; O, ovary; T, tube; A, vagina/rectovaginal space; B, uterosacral ligaments; C, rectum; and F, other locations, such as adenomyosis, bladder, intestines, and ureter). It uses a scale from 1 to 3 to indicate the extent of endometriosis in the following compartments (21, 22). A summary of the findings from laparoscopies performed in adolescent patients is provided in Table 1.

**Table 1:** A summary of the findings from laparoscopies performed on adolescents.

Case report (no.)	Age (years)	Distinctive feature (symptoms, laboratory findings)	Imaging (ultrasound)	Surgical observations in laparoscopy	rASRM classification	#ENZIAN classification	Postoperative treatment	Follow-up and treatment outcomes
1	19	Recurrent menstrual pain non-responding to pain management	No abnormalities	Red lesions on the peritoneum 3 cm	II	P1 O1/1 T1/1 A0 B1/1 C1	Dienogest 2 mg/day, 6 months	No recurrence
				Brown endometriosis on both ovaries <3 cm				
				Lesions on the rectouterine pouch with Allen–Masters windows				
		Laboratory test results—normal						
		Bowel endometriosis		Lesions on sacrouterine ligaments				
				Lesions on the rectum and sigmoid colon				
				Millet seed-like lesions on the large intestine				
2	15	Very high CA-125 level = 710 U/mL	Ovarian tumor	Red-flame lesions on peritoneum <3 cm	III	P1 O0/0 T2/2 A0 B1/1 C0	Dienogest 2 mg/day, 6 months	Regression of most endometriotic lesions (second-look laparoscopy)
				Ovaries with membranous-solid adhesions to the fimbriae of both fallopian tubes; conglomerate of adhesions				
		Menstrual pain -VAS 10 points						
		Bilateral hydrosalpinx		Fallopian tubes bilaterally thickened with adhesions of parietal peritoneum and greater omentum; fimbriae not visible, retracted into the conglomerate of adhesions; bilaterally overgrowth of the abdominal ostium				
				Adhesions in the rectouterine pouch with Allen–Masters windows				
				Sacrouterine ligaments thickened, with small foci of brown endometriosis <0.5 cm				
				Red and brown lesions on the serosa of the large intestine <1 cm				
3	17	Pelvic pain	No abnormalities	Red-flame lesions on peritoneum <3 cm	III	P1 O0/0 T1/3 A0 B2/0 C0	Dienogest 2 mg/day, 6 months	No recurrence
		Non-cyclic pain						
		Dyspareunia		Left ovary in adhesions with sigmoid colon				
				Left fallopian tube adherent to the abdominal wall				
				50 mL of blood-brown fluid in the rectouterine pouch				
				Left-side sacrouterine ligaments and parametrial shortening				
				Allen–Masters window in left perimetrium area				
				Pelvic organs shift and uterine adherence to anterior abdominal wall				
4	17	Membranous-like tissue endometriosis with chronic abdominal pain	Ovarian tumor	Red and clear membranous lesions on peritoneum >7 cm	II	P3 O0/0 T0/0	Dienogest 2 mg/day, 6 months	No recurrence
						A0 B0 C0		
				Red and clear membranous lesions on the rectouterine pouch				
				Red and clear membranous lesions on the intestinal surface				
				Peritoneal scarring, superficial red and clear lesions on the peritoneum, perimetrium and intestinal serosa				

### 2.1 Case report 1

**Figure 1:**
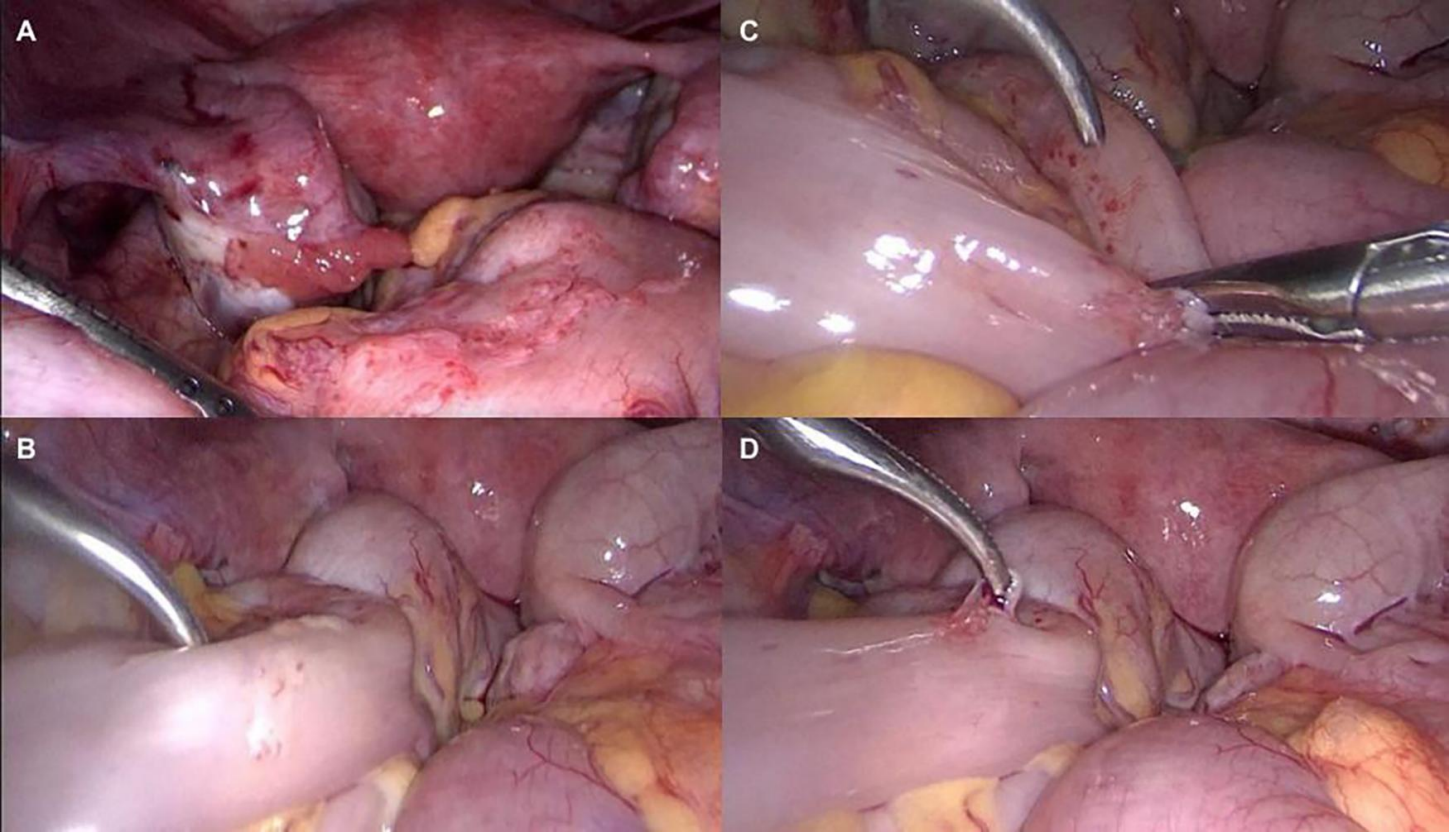
**(A–D)** Case 1: millet seed-like endometriotic implants on intestinal serosa.

A 19-year-old female patient presented with recurrent menstrual pain that did not respond to pharmacological treatment. Ultrasound examination revealed no abnormalities. The differential diagnosis prior to laparoscopy included other causes of secondary dysmenorrhea, pelvic inflammatory conditions, and bowel disorders. During diagnostic laparoscopy, approximately 150 mL of residual blood was found in the rectouterine pouch and red endometrial foci on the pelvic peritoneum. Endometriotic implants <1 cm in diameter were present on the surface of both ovaries and in the fallopian tubes, broad ligaments, uterosacral ligaments, and rectouterine pouch. Moving toward the patient's intestinal region, atypical lesions for adolescents were observed as millet seed-like endometriotic implants resembling vesicles on the surface of the colon, rectum, and cecum (Figures 1A–D). Endometriosis was confirmed and classified as stage II in rASRM score and P1 O1/1 T1/1 A0 B1/1 C1 in #ENZIAN. Postoperative treatment involved dienogest 2 mg daily for 6 months. The patient remained asymptomatic over a 1-year follow-up, during which she was regularly monitored. She remained asymptomatic, and the therapy was well-tolerated.

### 2.2 Case report 2

**Figure 2:**
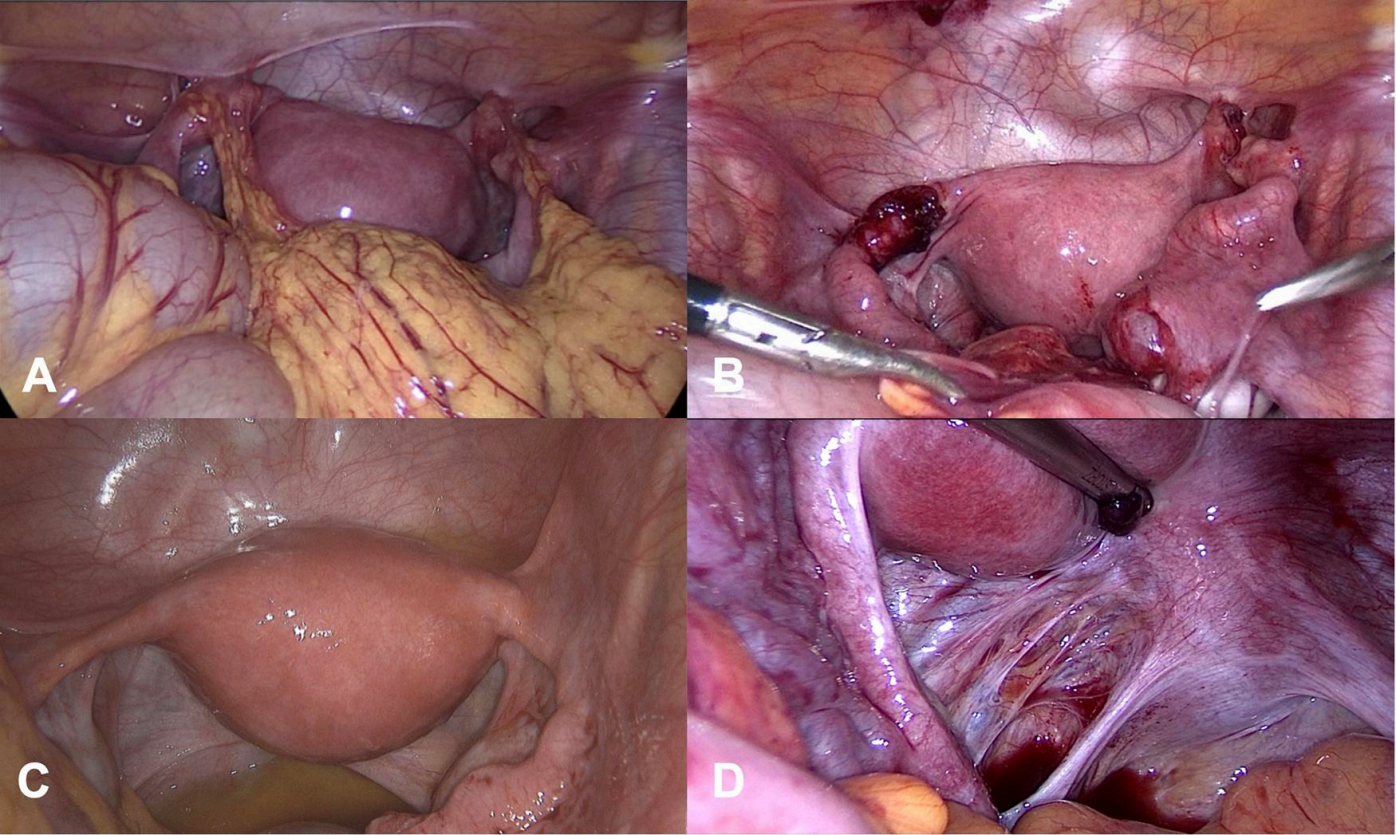
Findings from the laparoscopy. Case 2. **(A)** Symmetrical adhesions of the greater omentum with fallopian tubes and anterior abdominal wall, bilateral periadnexal adhesions. **(B)** Bilateral hydrosalpinx visible after adhesion dissection. **(C)** Second-look laparoscopy. Case 3: **(D)** Allen–Masters windows.

A 15-year-old female patient reported menstrual pain at 10/10 points on the visual analog scale (VAS). An ultrasound scan performed 1 month prior to admission suggested the presence of either ovarian endometriomas or a neoplastic tumor. Serum cancer antigen 125 (CA-125) was 710 U/mL (reference range 0–35 U/mL). Thus differential diagnosis included not only the tumor but also pelvic inflammatory disease. She had never been sexually active. The laparoscopy revealed widespread adhesions distributed in many locations: between the uterus and adnexa, between the greater omentum and both fallopian tubes, and between the fallopian tubes and peritoneum (Figure 2A). The right uterosacral ligament was shortened, causing distortion of the uterus on the right. Blood collection was visible in the rectouterine pouch. After the fallopian tubes had been mobilized from adhesions, occlusion of both abdominal ostia was noticed. The walls of the fallopian tubes were thickened. The ampullae of both fallopian tubes were dilated, consistent with hydrosalpinx. Bilateral hydrosalpinx (Figure 2B) is also uncommon in sexually inactive adolescents but is more likely to appear in adolescents with inflammatory disease. Adhesions were removed, multiple biopsies were taken from endometriotic implants rich in membranous-like tissue endometriosis, and the collected fluid was analyzed. The hydrosalpinx was not removed due to the patient's age; instead, a conservative treatment approach was chosen.

The patient's condition severity was evaluated as grade III according to the rASRM, and #ENZIAN was assessed as P1 O0/0 T2/2 A0 B1/1 C0. Postoperative treatment involved dienogest 2 mg daily for 6 months. CA-125 after 6 months of treatment was within normal limits. However, due to uncertainty, the patient underwent a second-look laparoscopy after 6 months, confirming regression of most endometriotic lesions (Figure 2C). Single endometriotic foci on the fallopian tubes were seen. Shortening of the right uterosacral ligament and delicate distortion of the uterus to the right remained. The patient reported no complaints.

### 2.3 Case report 3

A 17-year-old female patient was referred for diagnostic laparoscopy due to suspected endometriosis. She reported non-cyclic pelvic pain, abdominal pain, and dyspareunia. The ultrasound did not reveal any abnormalities. Substantial shortening of the left parametrial and uterosacral ligaments was detected. In the left parametrium area, Allen–Masters windows were observed (Figure 2D). Laparoscopy revealed a flattened retropubic space, a notable pelvic organ shift to the left, and uterine adherence to the anterior abdominal wall. The patient was classified as rASRM stage III; #ENZIAN P1 O0/0 T1/3 A0 B2/0 C0. Postoperative treatment involved dienogest 2 mg daily for 6 months. The patient remained free of recurrence and reported good tolerance of the ongoing treatment.

### 2.4 Case report 4

**Figure 3:**
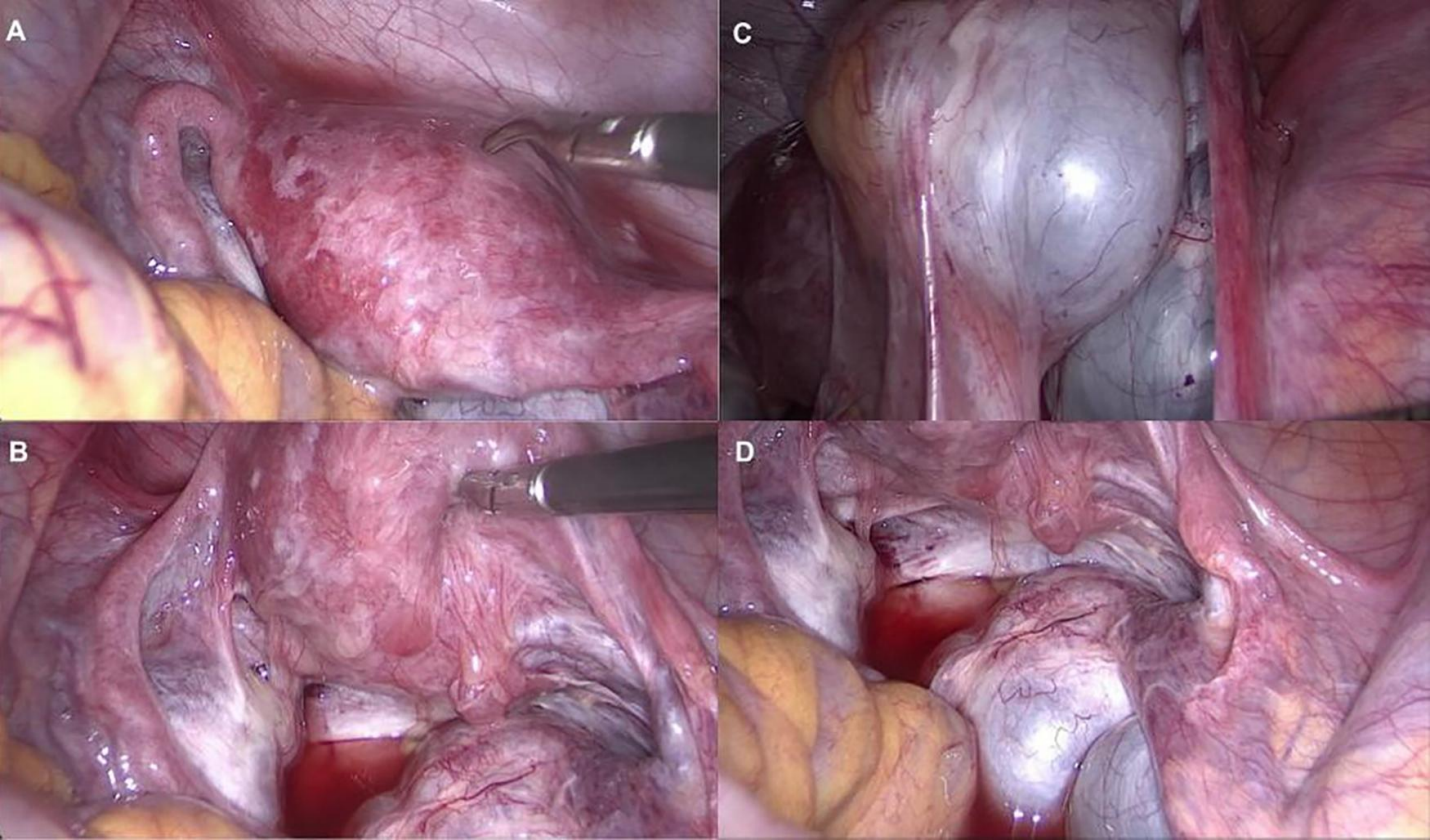
Case 4. **(A, B)** Clear lesions. **(C)** Adhesions of the ovary **(D)** Mucinous cyst adenoma of the left ovary.

A 17-year-old female patient underwent a diagnostic laparoscopy prompted by strong suspicions of an ovarian tumor based on ultrasound examination. The main complaint reported by the patient was abdominal pain. The initial differential diagnosis mainly focused on ovarian pathology. Laparoscopy confirmed the presence of a left ovarian tumor and additionally revealed endometriosis, classified as rASRM II, #ENZIAN P3 O0/0 T0/0 A0 B0/0 C0 with peritoneal scarring, superficial red and clear lesions on the peritoneum, perimetrium, and intestinal serosa (Figures 3A–C). Histopathological examination confirmed the presence of ovarian mucinous cystadenoma in the enucleated tumor (Figure 3D). Postoperative treatment involved dienogest 2 mg daily for 6 months. No recurrence has been detected, and treatment tolerance is satisfactory.

In all cases, a diagnostic and therapeutic laparoscopy was performed due to the high possibility of endometriosis in these adolescent patients (aged 15–19 years old). The surgical procedures were performed under general anesthesia, using standard laparoscopic access. The surgery aimed to release adhesions, access abnormalities, and confirm the diagnosis. Postoperatively, all patients received the same treatment, i.e., dienogest 2 mg daily for 6 months. Both the surgical treatment and subsequent pharmacological treatment were well-tolerated, with no adverse and unanticipated events. All patients remain free of recurrence and report good tolerance of the ongoing treatment, representing a therapeutic success despite the moderate stage of endometriosis.

## 3 Discussion

We presented cases with a wide spectrum of laparoscopic images and symptoms of adolescent endometriosis. Moreover, the majority of the findings are atypical and not described among adults, as well as uncommon among pediatric and adolescent patients. In the first case, we described extragenital endometriosis on the bowel surface. These lesions cannot be identified by ultrasound imaging and are not present in literature reviews (9).

In adult women, bowel endometriosis is the most common site of extragenital endometriosis and mainly affects the rectum and sigmoid colon (23, 24). Pain signaling is enhanced by local neurogenesis and recruitment of nerve fibers in the area of endometriosis-induced inflammation. Endometriotic lesions dominate in the most innervated layers of the colon with extensive endoneurial and perineurial invasion. Thus, it has been proposed that nerves may play a key role in the spread of the disease (25). Bowel endometriosis is closely linked to fibrosis, angiogenesis, and local invasion, which results in the formation of nodules leading to discomfort and pain-related symptoms (26). The nodules found on the intestinal surface are classified as symptoms of DE. The millet seed-like endometriotic implants observed on the surface may represent the early appearance of bowel endometriosis associated with severe gastrointestinal symptoms during menstruation. Allen–Masters windows, observed in the rectouterine pouch (12), are atypical defects of the peritoneum. Researchers discovered that these findings are related to endometriosis-driven scarring, duplication, and reduplication of the peritoneal areas, which is a result of cyclic damage to ectopic endometrium (27). The second case is more likely to be seen in adult female patients than in adolescents. The high blood levels of CA-125 accompanying the abnormal ovarian ultrasound results created suspicion of an ovarian tumor in early diagnostic hypotheses. The combination of clinical presentation increases the risk of misinterpretation. Furthermore, the patient had a very uncommon finding in adolescents—hydrosalpinx. Only one case report linking hydrosalpinx to endometriosis has been identified in the literature (28). Merlini et al. (29) presented bilateral hydrosalpinges as a surgical complication in a 17-year-old female patient. Clinicians should always be aware of the increased risk of tubal or adnexal torsion in such cases and be prepared for emergency surgery if symptoms indicate it (30). Follow-up laparoscopy supported the efficacy of conservative treatment over invasive surgery in adolescents. The patient experienced early menarche (11 years old), raising the possibility of a correlation between age at menarche and the severity of anatomical changes (31).

Despite hormonal or surgical treatment, pain may persist in some patients; one potential explanation is coexisting adenomyosis (32, 33). Adenomyosis, characterized by ectopic endometrial glands and stroma within the myometrium, leading to hyperplasia and hypertrophy of surrounding smooth muscle, is a common uterine disorder associated with heavy menstrual bleeding, dyspareunia, dysmenorrhea, pelvic pain, and infertility. The coexistence of adenomyosis with endometriosis has been reported in 20%–75% of cases, particularly in patients with chronic pelvic pain or persistent symptoms after surgery (34). The prevalence of adenomyosis (based largely on hysterectomy series) ranges from 14% to 57% (5). Some studies have indicated that severe endometriosis is particularly associated with diffuse adenomyosis, suggesting shared pathophysiological mechanisms between these diseases (33).

During the diagnostic work-up for concomitant adenomyosis, recommended steps include abdominal and pelvic examination (often revealing uterine enlargement and tenderness) and ultrasound, transvaginal in sexually active patients or transrectal otherwise, with three-dimensional and volume-contrast techniques to aid differentiation from fibroids. MRI can be used when ultrasound is inconclusive or not feasible, and CT only in selected circumstances after ultrasound (35). Choosing the therapeutic approach in adolescents should prioritize fertility preservation; hormone therapy is first-line, with surgery considered individually based on symptom severity, response to treatment, and disease extent (32). The uterine anatomy presented in the third case was altered due to inflammation and adhesions caused by endometriosis, which resulted in an advanced stage according to the rASRM classification. In a young patient, pelvic organ shift is an uncommon presentation of the disease. Assessment remains limited without laparoscopy, and ultrasound may not be sufficient to detect these findings. To the best of our knowledge, such a marked shortening of sacrouterine and parametrial ligaments and such an advanced pelvic organ shift have not been reported in adolescents. The fourth case highlights the challenges of diagnosing endometriosis as endometriosis was confirmed alongside the ovarian tumor, which suggests potential co-occurrence of these conditions, leading to being overlooked during initial examination. The fourth case is an example of the coexistence of endometriosis with another disease and requires a multidimensional therapeutic approach, including both endometriosis and ovarian tumor management. The prevalence of ovarian masses in the pediatric population is estimated at 2.6 per 100,000 (36). In comparison to endometriosis (10%–15% of adolescents), it is notably less common (1). Case studies primarily report menstrual disorders and abdominal pain in young patients with ovarian tumors (37). The ovarian endometriomas in adolescents are considered rare, although in one prospective study, ovarian endometriomas were found in 20.7% of the 345 patients (38).

Adolescents and young women commonly experience dysmenorrhea or pelvic pain, which may be early signs of endometriosis. The first step is to collect a comprehensive medical interview. Particular attention should be paid to menstrual and psychosocial aspects, including family history of endometriosis, early menarche, dyspareunia, abnormal uterine bleeding (heavy or irregular bleeding), midcycle or acyclic pain, inadequate response to empirical analgesics or hormonal therapy, gastrointestinal and genitourinary symptoms, impairment of daily activities, and school absenteeism. It is also important to ask about fatigue, nausea, and diarrhea and to address the common social normalization of severe menstrual pain (33, 39). Carrying out a questionnaire provides significant help in identifying and assessing symptoms of the disease. Physical examination of the abdomen and pelvis is essential in the initial assessment of adnexal masses, nodules, and abnormalities of the uterosacral ligaments (e.g., retraction or thickening). Non-invasive imaging is the first-line diagnostic approach: in non-sexually active patients, transabdominal and/or transrectal ultrasound may be used. MRI is considered a second-line modality, useful when ultrasound is inconclusive or to map deep disease. Determining whether dysmenorrhea is primary or secondary and distinguishing gynecologic from non-gynecologic causes of pelvic pain are essential (33).

There are numerous theories and pathogenetic explanations for the onset of endometriosis. Sampson's theory (40) of retrograde menstruation is considered a main mechanism of endometriosis development nowadays. In accordance with this theory, blood backflows into the peritoneal cavity from the uterus via the fallopian tubes. However, the incidence of lesions visibly consistent with endometriosis in premenarchal girls with no obstructive anomalies supports the idea that some cases of endometriosis may result from other etiologies (41). The second case was unusual because it remains unclear why such severe lesions developed at such a young age. That observation favors the thesis of an early-onset endometriosis origin. It is the opposite of the widely distributed belief about the peak in the occurrence of endometriosis in the postmenarchal population (42–44). Habiba et al. (45) revised theories of the early onset of endometriosis and hypothesized the link between neonatal uterine bleeding and early onset of endometriosis. Neonatal uterine bleeding, similarly to the retrograde menstruation theory, can be related to backflow to the pelvic cavity and seeding endometrial cells with differentiating and proliferating abilities. It has been proposed that embryonic Müllerian rests may be the start point of early-onset endometriosis. Based on this theory, endometriosis is asymptomatic before menarche due to a lack of estrogen at that time. The mechanism proposed in embryogenetic theory is based on residual embryonic cells of Wolffian or Müllerian ducts, which may develop into endometriosis lesions in response to estrogen. That theory is postulated when the endometriosis is found during the autopsy of human fetuses (46, 47). The biochemical and macroscopic difference between endometriosis lesions and endometrium can be explained by the genetic–epigenetic theory (48), which postulates that endometriosis lesions begin their development only after a cumulative number of genetic or epigenetic incidents have exceeded a certain threshold, changing the endometrial cell into an endometriotic cell, as supported by the differences in lipid profiles (48) and gene expression (49). Alternatively, widely accepted theories propose that endometrial cells can appear in extra-pelvic organs through different mechanisms: Halban’s theory of benign metastasis suggests hematogenous or lymphatic dissemination, while Meyer’s theory attributes endometriosis to be an effect of metaplasia of the coelomic epithelium (50). Another theory evaluates stem cell recruitment either from endometrial tissue or from bone marrow sources, or potentially from both (51–55). It is crucial to expand diagnostics especially for the pediatric population, which remains inadequately screened. Therefore, Koninckx et al. (56) suggested considering the diagnosis of endometriosis and potential preventive measures early in the adolescent daughters of women with endometriosis.

A comprehensive evaluation of CA-125 across 19 prospective and 3 retrospective observational studies, including 3,626 participants with histologically proven endometriosis, revealed a specificity of 93% and a sensitivity of just 52% for the condition. Evidence indicates that CA-125 may serve as a screening marker in symptomatic individuals; nevertheless, its limited sensitivity implies that a negative result does not exclude the presence of endometriosis, and a positive result may cause anxiety for the patient and increase the risk of overtreatment. As a result, studies suggest that CA-125 should not be used routinely for the diagnosis of endometriosis (13). Perhaps when taking into consideration all the abovementioned specific clinical features and differences between adult and adolescent endometriosis, this may indicate the need for a future evaluation toward establishing international pediatric and adolescent endometriosis guidelines. Several national obstetrics and gynecology societies, such as the Section of Pediatric and Adolescent Gynecology of the Polish Society of Gynecologists and Obstetricians, have already developed and published their own clinical guidelines (57). Diagnostics delay in adolescent populations has a multifaceted origin. It stems not only from the absence or mildness of symptoms but also from the reluctance to perform endometrial biopsies in precoital patients. Endometriosis that remains untreated or is diagnosed late is a significant risk factor for infertility and for gestational complications, including preterm birth, premature rupture of membranes, and stillbirth (13, 58).

The diagnostic approach in this case had several notable strengths. A complete and well-conducted patient history supplied crucial data on symptom onset, timeline, and severity. In adolescents, non-specific symptoms such as dysmenorrhea and gastrointestinal disorders affect a large proportion of patients with endometriosis, which should encourage clinicians to include endometriosis in the differential diagnosis. Particular attention should be paid to digestive problems in the diagnostic process, as the symptoms of bowel endometriosis. It is important to notice the everyday life problems, including reported issues with school/work absence or physical activity. Detailed laparoscopic evaluation provided detection of atypical clinical presentation of endometriosis, such as serosal lesions, organ shift, peritoneal adhesions, and hydrosalpinx. These findings highlight that the presence of non-specific features may mask endometriosis in adolescents, leading to missed diagnosis, and may suggest a more aggressive onset of the disease in young girls in comparison with adult women. However, a limitation is that the case series includes a limited number of patients, reducing the generalizability of the findings. There is a need for further studies and case collections to expand knowledge of clinical variations of adolescent endometriosis.

## 4 Patient perspective

The formation of a database of adolescent endometriosis cases could improve epidemiological and imaging analysis of its uncommon variants. Developing a specific adolescent classification of endometriosis may be beneficial for diagnostic efficiency. Early detection of the disease would enable early treatment onset and consequently a substantial reduction of long-term implications. We believe that lesions described above can be included in future diagnostic guidelines for endometriosis in children and adolescents and that it reflects the global paradigm shift toward an approach focused on the early detection of prodromal symptoms of endometriosis. It would not only improve the dismal statistics of underdiagnosed cases but also significantly mitigate the prevalence of absenteeism in young individuals affected by this condition.

## Data Availability

The original contributions presented in the study are included in the article/Supplementary Material; further inquiries can be directed to the corresponding author.
